# Diagnostic value of combined detection of FeNO and pulmonary function indexes in children with cough variant asthma

**DOI:** 10.1097/MD.0000000000044558

**Published:** 2025-09-19

**Authors:** Minghang Yang, Xiaofang Zhang, Xiaozheng Duan, Yina Qiao, Xiaochun Feng

**Affiliations:** a Changchun University of Chinese Medicine, Changchun, Jilin Province, China; b First Affiliated Hospital to Changchun University of Chinese Medicine, Changchun, Jilin Province, China; c Second Affiliated Hospital to Shaanxi University of Chinese Medicine, Xian, Shaanxi Province, China; d Third Affiliated Hospital to Changchun University of Chinese Medicine, Changchun, Jilin Province, China.

**Keywords:** children, cough variant asthma, exhale nitric oxide, pulmonary function

## Abstract

Cough variant asthma (CVA) is a special type of asthma and one of the primary causes of chronic cough in children. The aim of this study was to investigate the diagnostic value of combined detection of fractional exhale nitric oxide (FeNO) and pulmonary function parameters in children with CVA. A retrospective study was conducted on 120 children with CVA. Sixty children (36 boys and 24 girls, age = 4.97 ± 0.928 years) with CVA admitted to the pediatric clinic of the hospital from June 2021 to June 2022 were selected for the CVA group, and 60 children (32 boys and 28 girls, age = 4.90 ± 0.706 years) with chronic cough without CVA who visited the pediatric clinic of the hospital during the same period were selected for the NCVA group. FeNO and pulmonary function parameters (maximum mid-expiratory flow [MMEF], maximal expiratory flow 25 [MEF25], maximal expiratory flow 50 [MEF50], maximal expiratory flow 75 [MEF75]) were compared between the 2 groups. Receiver operating characteristic curve was used to analyze the diagnostic value of FeNO and pulmonary function parameters for CVA, and the correlation between the severity of CVA and the above parameters was analyzed. The FeNO (41.90 ± 3.111 ppb) level in the CVA group was significantly higher than that in the NCVA group (19.98 ± 6.231 ppb), and the difference was statistically significant (*P* = .000). The MMEF, MEF75, MEF50, and MEF25 levels were lower than those in the NCVA group, and the differences were statistically significant (*P* = .000). Receiver operating characteristic curve analysis results showed that the area under the curve of FeNO, MMEF, MEF75, MEF50, and MEF25 in the diagnosis of CVA was 0.726, 0.638, 0.611, 0.709,and 0.734, respectively, and the area under the curve of the combined detection of the 5 indicators was 0.901. The combined detection has higher sensitivity and specificity, which can significantly improve the diagnostic efficacy of CVA.

## 1. Introduction

Cough variant asthma (CVA) is a special type of asthma with chronic cough as the main symptom, without obvious signs such as shortness of breath, which often occurs at night or in the early morning and is one of the common causes of chronic cough in children.^[1]^ It was induced due to factors such as cold air, haze, irritating odor, or exercise to aggravate the symptoms, mostly accompanied by a personal or family history of eczema or allergic history.^[2]^

In recent years, the incidence of CVA has been increasing year by year due to the environmental pollution, the opening of COVID-19 and the increase of atypical pathogen infection, which account for up to 35.1% in Asia.^[3]^ Its diagnostic methods have attracted much attention in the medical community.^[4]^ Eosinophilic airway inflammation is an important pathological feature of CVA. The confirmation of CVA often requires induced sputum cytology as well as bronchial provocation tests.^[5]^ However, the induced sputum cytology and bronchial provocation test not only require high professional technical requirements, but also require the active cooperation of children, which is difficult to implement in clinical work. Fractional exhaled nitric oxide (FeNO) is a noninvasive, sensitive, and convenient biomarker of airway inflammation that is commonly used to monitor eosinophilic lower respiratory tract inflammation and is easy to perform.^[6]^ Pulmonary function test is one of the necessary examination methods for respiratory system diseases. Maximum mid-expiratory flow (MMEF), maximal expiratory flow 75 (MEF75), maximal expiratory flow 50 (MEF50), and maximal expiratory flow (MEF25) can reflect the functional status of small airways. Pulmonary function indexes have important guiding significance for early diagnosis of disease severity and medication evaluation. Pulmonary function test can reflect the state of airway function comprehensively on the basis of FeNO test.^[7]^

The initial manifestations of CVA are very similar to those of other chronic coughs, especially postinfectious coughs. If FeNO and pulmonary function tests are not performed, the initial medication may cause the abuse of antibiotics.^[8]^ These children with CVA are not diagnosed until the middle to late stages when clinical symptoms become obvious, resulting in missing the optimal treatment window. Feng-Jia et al^[9]^ retrospectively analyzed 150 patients with CVA and found that small airway function was significantly lower compared with patients without CVA, so the results of this study are consistent with previously published results. Liu et al^[10]^ reported that FeNO test combined with impulse oscillometry test improves the diagnostic sensitivity and specificity of small airway dysfunction in asthmatic patients. However, there are few reports on combining the 2. In our study, the combination of FeNO and small airway function parameters in pulmonary function testing was utilized to reflect the dual pathological mechanisms underlying CVA. FeNO primarily evaluates eosinophilic airway inflammation levels, while pulmonary function parameters such as MMEF, MEF50, and MEF25 quantify small airway obstruction. This integrated approach captures both the inflammatory status and functional impairment of airways, offering a comprehensive assessment of CVA pathophysiology. Moreover, FeNO reflects type 2 inflammatory pathways driven by cytokines,^[11]^ whereas small airway parameters correlate with structural and functional abnormalities in distal airways. Such synergistic evaluation not only improves early detection of subclinical inflammation but also aids in monitoring therapeutic responses to inhaled corticosteroids. Some studies^[12]^ suggest that about 30% of the CVA population eventually develops typical asthma due to lack of regular treatment. In this study, FeNO combined with pulmonary function tests were used to predict the occurrence of small airway disease, and precise medication was conducive to the rehabilitation of airway and pulmonary function and prognosis in children with CVA.

FeNO and pulmonary function test are noninvasive and easily implementable diagnostic tools, particularly suitable for pediatric populations. It solves the problem that the traditional examination (such as bronchial excitation test) is difficult to popularize. The hypothesis is that FeNO combined with pulmonary function tests can improve the diagnostic sensitivity and specificity of CVA in children through complementary mechanisms, then this study can explore the diagnostic value of FeNO and lung function parameters for CVA in children, thereby improving the detection rate of CVA.

## 2. Method

Sixty children with CVA admitted to the Department of Pediatrics of the hospital were selected as the observation group, and another 60 children with chronic cough of non-CVA (NCVA) in the same period were selected as the control group. The levels of FeNO and pulmonary function indexes (MMEF, MEF25, MEF50, MEF75) were compared between the 2 groups, and the diagnostic value of FeNO and pulmonary function indexes for CVA was analyzed using receiver operating characteristic (ROC) curve.

### 2.1. Patients and controls descriptions

In the CVA group, 60 children (26 males and 24 females) with CVA admitted to the pediatric clinic of the hospital from June 2021 to June 2022 were selected. In the NCVA group, 60 children (32 males and 28 females) with chronic cough without CVA who visited the hospital at the same time were selected. In addition to routine clinical visits, cases were also recruited through poster advertisements. The included cases were all examined during the day and no evening tests were performed.

Inclusion criteria: patients were eligible for inclusion if they met the diagnostic criteria for CVA and chronic cough as outlined in the Guidelines for the Diagnosis and Prevention of Bronchial Asthma in Children and were aged 12 years or younger.

Exclusion criteria: exclusion criteria included combined allergic diseases, malignant tumors, contraindications to pulmonary function tests, organic diseases such as heart and brain, combined with other sites of infection, systemic connective tissue diseases, metabolic, endocrine diseases, and mental illness. Immunosuppressants used before the trial were exclusion criteria. The age requirements specified in the exclusion criteria align with those defined in the inclusion criteria. All children’s families gave informed consent and signed informed consent. This study was reviewed and approved by the medical ethics committee of our hospital.

### 2.2. FeNO and pulmonary function parameters

FeNO was measured using the electrochemical sensor-based NIOX MINO system (Aerocrine AB, Solna, Sweden) in our lab. The FeNO levels were measured in all study subjects and performed in strict accordance with the instructions. At the beginning of the test, the child was asked to exhale outwards, try to empty the air in the lung. Then firmly secure the mouthpiece and inhale forcefully until airflow ceases, then apply slight negative pressure to ensure complete inhalation without airflow interruption. The FeNO results were obtained as ppb. The device provides FeNO measurements at an exhalation flow rate of 50 mL/s, with a specified accuracy of ±3 ppb for values <30 ppb and ±10% for values ≥30 ppb.

Pulmonary function indicators of all study subjects were measured using a lung function instrument (Master Screen, Jaeger, Becton, Dickinson and Company, Höchberg, Germany). The lung function instrument needs to be calibrated daily. Before testing, the height and weight of the child had been measured in advance, and basic information such as sex, age, or month age should be recorded. The examination should be conducted 1 to 2 hours after eating, to avoid obvious abdominal distension, remove nasopharyngeal secretions, and keep the upper airway unobstructed. During the measurement period, all subjects were seated and instructed to breathe normally through the mouthpiece, with their nose clamped with a nose clip and their cheeks supported by the operator’s hand. The child breathed spontaneously and calmly. The data were collected after the baseline was stable. The sampling time was about 30 seconds.

The detection of pulmonary function parameters: pulmonary function parameters were measured in all subjects, including MMEF, forced expiratory flow at 25% of the forced vital capacity (MEF25), forced expiratory flow at 50% of the forced vital capacity (MEF50), and forced expiratory flow at 75% of the forced vital capacity (MEF75). All subjects were tested twice and the mean value was taken.

### 2.3. Statistical analysis

Data were analyzed using IBM SPSS Statistics 23.0 (IBM Corporation, Armonk). The measurement data were analyzed by Kolmogorov–Smirnov method for normality test. The measurement data conforming to normal distribution were described as mean ± standard deviation $\left(\bar{x}\pm s\right)$. The measurement data of normal distribution between the 2 groups were compared by independent sample *t*-test or Mann–Whitney *U* test. Categorical variables were presented as n (%), using the chi-square test. Cohen *d* was used as a measure of effect size for outcomes that were examined by comparing the mean values; Cohen *d* of 0.2, 0.5, and 0.8 define a small, medium, and large effect size. ROC curve was used to analyze the diagnostic value of MMEF, MEF75, MEF50, and MEF25 combined with FeNO levels in children with CVA, and *Z* test was used to compare the area under the ROC curve between combined diagnosis and single diagnosis, with a significant difference at *P* < .05.

## 3. Results

### 3.1. Characteristics of the patients

There was no significant difference in age, height, weight, course of disease, and other general data between the CVA group and the NCVA group (*P* > .05). The FeNO and pulmonary function index levels in the CVA group and the NCVA group were significantly higher than those in the NCVA group, and the MMEF, MEF75, MEF50, and MEF25 levels were lower. See Table 1 for details.

**Table 1:** Characteristics of CVA and NCVA (N = 120) $(\bar{x}\pm \;s)$.

Variable	CVA	NCVA	*t*	*P*	Cohen *d*
Age (yr)	4.97 ± 0.928	4.90 ± 0.706	0.379	.705	0.0849
Height (cm)	109.93 ± 4.941	112.48 ± 8.607	−1.501	.137	0.3634
Weight (kg)	18.68 ± 2.775	19.63 ± 4.523	−1.054	.295	0.2532
Course of disease (week)	6.80 ± 2.709	6.92 ± 2.593	−0.198	.843	0.0453
FeNO (ppb)	41.90 ± 3.111	19.98 ± 6.231	18.131	<.001	4.4511
MMEF (% predicted)	48.17 ± 3.119	60.90 ± 9.619	−7.050	<.001	1.7803
MEF75 (% predicted)	69.73 ± 2.303	80.70 ± 1.522	−26.995	<.001	5.62
MEF50 (% predicted)	58.93 ± 1.893	71.07 ± 2.400	−24.168	<.001	5.6167
MEF25 (% predicted)	50.53 ± 1.978	63.40 ± 1.834	−30.566	<.001	6.7475

CVA = cough variant asthma, FeNO = fractional exhaled nitric oxide, MEF25 = forced expiratory flow at 25% of the forced vital capacity, MEF50 = forced expiratory flow at 50% of the forced vital capacity, MEF75 = forced expiratory flow at 75% of the forced vital capacity, MMEF = forced expiratory flow between 25 and 75%, NCVA = non-cough variant asthma, ppb = parts per billion.

### 3.2. ROC curve analysis of FeNO and pulmonary function indexes in the diagnosis of CVA

**Figure 1.**
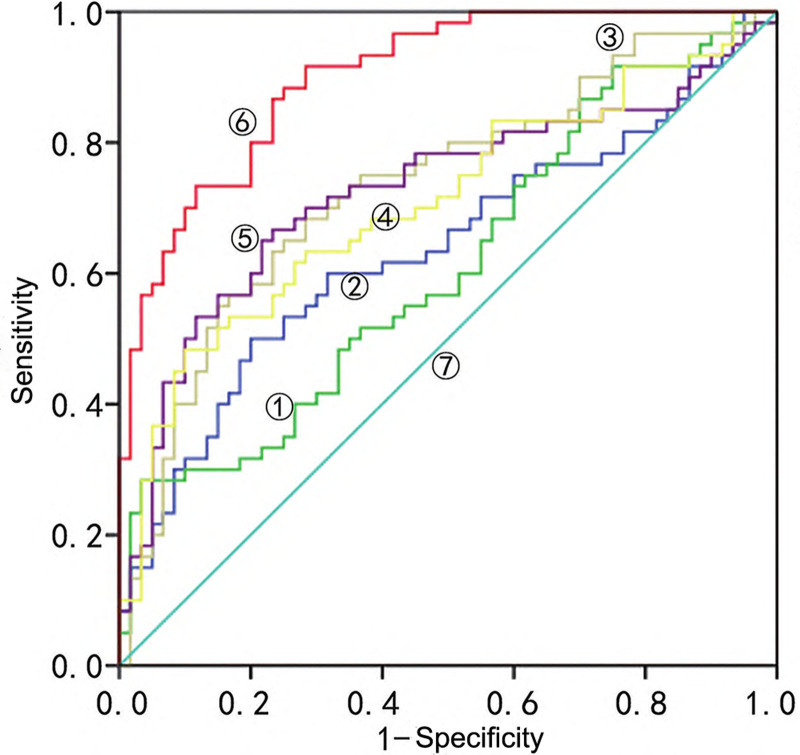
ROC curve analysis of FeNO and pulmonary function indexes in the diagnosis of CVA. ①, MEF75; ②, MMEF; ③, MEF25; ④, MEF50; ⑤, FeNO; ⑥, five combined; ⑦, reference line. CVA = cough variant asthma, FeNO = fractional exhale nitric oxide, MEF25 = maximal expiratory flow 25, MEF50 = maximal expiratory flow 50, MEF75 = maximal expiratory flow 75, MMEF = maximum mid-expiratory flow, ROC = receiver operating characteristic.

The results of ROC curve analysis showed that the area under the curve (AUC) of FeNO, MMEF, MEF75, MEF50, and MEF25 in the diagnosis of CVA was 0.726, 0.638, 0.611, 0.709 and 0.734, respectively. The AUC of the combined detection of the 5 indicators in the diagnosis of CVA was 0.901. See Table 2 and Figure 1 for details.

**Table 2:** Optimal cutoff values for the prediction of CVA.

Variable	Cutoff	SE	*P*	AUC (95% CI)	Sensitivity	Specificity
FeNO (ppb)	39.83	0.048	.001	0.726 (0.638–0.802)	0.765	0.676
MMEF (% predicted)	64.23	0.051	<.001	0.638 (0.545–0.723)	0.721	0.499
MEF75 (% predicted)	99.37	0.052	<.001	0.611 (0.519–0.698)	0.966	0.282
MEF50 (% predicted)	78.14	0.048	<.001	0.709 (0.618–0.789)	0.899	0.481
MEF25 (% predicted)	61.48	0.046	<.001	0.734 (0.646–0.811)	0.716	0.693
Five Combined	–	0.026	<.001	0.901 (0.832–0.949)	0.922	0.868

AUC = area under the curve, CI = confidence interval, CVA = cough variant asthma, FeNO = fractional exhaled nitric oxide, MEF25 = forced expiratory flow at 25% of the forced vital capacity, MEF50 = forced expiratory flow at 50% of the forced vital capacity, MEF75 = forced expiratory flow at 75% of the forced vital capacity, MMEF = forced expiratory flow between 25% and 75%, ppb = parts per billion, SE = standard error.

## 4. Discussion

Cough is a common cause of treatment in pediatric clinics and prolonged treatment of cough not only reduces the quality of life of children, but also brings a heavy economic burden to families and society.^[13]^ In this study, FeNO and pulmonary function parameters were used to diagnose the CVA. The sensitivity and specificity of single index and combined detection of 5 indicators were compared. The results of this study showed that FeNO levels were higher and MMEF, MEF75, MEF50, and MEF25 levels were lower in the CVA group than in the NCVA group (*P* < .05), indicating that FeNO, MMEF, MEF75, MEF50, and MEF25 may be effective indicators for distinguishing CVA from NCVA.

NO in the airways is a biological mediator produced by l-arginine catalyzed by nitric oxide synthase in airway epithelial cells.^[14]^ When type II inflammation is induced by external stimuli in the airways, it can lead to increased expression of inducible nitric oxide synthase in airway epithelial cells, which catalyzes l-arginine to produce a large amount of NO excreted from the airways. Clinically, FeNO testing can be used for the diagnosis of asthma and chronic cough. Previous studies have found significantly higher FeNO levels in patients with cough-variant asthma as compared with other chronic cough patients.^[15]^ However, due to the optimal threshold of FeNO and the differences in diagnostic sensitivity and specificity, the diagnostic role of FeNO in cough variant and asthma is somewhat controversial. The results of this study showed that the sensitivity and specificity of FeNO for CVA were 0.765 and 0.676, respectively, with a cutoff value of 39.83 ppb. Sato et al^[16]^ found that FeNO was higher in children with CVA than in those with chronic cough of other etiologies, and the sensitivity and specificity of its diagnosis were 0.792 and 0.913, respectively, if FeNO measurements were ≥38.8 ppb. This showed that FeNO levels can better reflect airway class II inflammation and predict glucocorticoid treatment outcomes.^[17]^ FeNO detection process is completed in spontaneous breathing calm state, without special active cooperation requirements. It is commonly used in the diagnosis of a variety of respiratory diseases in children. The American Thoracic Society/European Respiratory Society issued a joint statement, confirming the importance of FeNO as a marker of airway inflammation reflecting eosinophilic airway inflammation and evaluating responsiveness to glucocorticoid therapy.^[18]^ Many clinical experts also recommend this detection technique for the diagnosis of chronic cough, bronchial asthma in children, and wheezing in infants.^[9,19]^

Routine spirometry is an important method to assess and diagnose the etiology of chronic cough in children. Chen et al^[20]^ retrospectively analyzed 150 adult patients showing that the most common feature of CVA is airway dysfunction, especially small airway dysfunction. Tian et al^[21]^ found that small airway resistance was significantly higher in children with CVA than in healthy children by impulse oscillometry, but their study did not include an analysis of FeNO measurements. The conventional lung function parameters used to assess small airways in the study were included in MMEF, MEF25, and MEF50. Because of physiological structure, vital capacity, tidal volume, minute ventilation, and gas diffusion capacity are smaller in children than in adults, while airway resistance is greater in children than in adults.^[22]^ The results showed that the levels of MMEF, MEF75, MEF50, and MEF25 in children with CVA were significantly lower than those in children with chronic cough, indicating that the main feature of CVA was small airway dysfunction. Wang et al^[23]^ showed that pulmonary function tests can be used as indicators for screening CVA in patients with chronic cough. The small airway function was more significantly reduced similar to the results of this study. These decreases may reflect pathological processes such as airway smooth muscle contraction, increased mucus secretion, or structural changes in small airways, which together contribute to the limitation of expiratory flow. It suggests that the small airway index has certain diagnostic value for the disease. The AUC of FeNO combined with small airway parameters was 0.901 which was significantly higher than that of FeNO or small airway parameters alone. Chen et al^[20]^ showed an AUC of 0.87 for FeNO combined with MMEF and 0.873 for FeNO combined with MEF50. This result is also higher than previous studies. The results showed that the combined detection was superior to single parameter in the diagnosis of CVA (*P* < .05). It suggested that the pathogenesis of CVA is usually caused by small airway injury, which may be related to persistent airway inflammation, causing mucosal swelling and causing small airway function injury. The results of ROC curve analysis showed that pulmonary function parameters had certain clinical value in the diagnosis of CVA. The results showed that the AUC of FeNO combined with pulmonary function indexes (MMEF, MEF75, MEF50, MEF25) in the diagnosis of CVA was higher than that of single index. It indicated that the combined detection of the 5 indexes has a higher diagnostic value for CVA.

Unlike previous studies in adults, this study mainly focused on pediatric cases, and due to the large age differences and different respiratory physiological functions of children, it is necessary to consider whether the children still have obstructive lesions in combination with other indicators. Because the combination of these measures significantly improves diagnostic value, it may be helpful in the identification of CVA in children with chronic cough.

In clinic, the initial clinical presentation of CVA closely resembles that of other chronic cough, particularly postinfection cough. If FeNO and pulmonary function tests are not performed, the initial diagnosis is not clear and medication can cause abuse of antibiotics, resulting in that children with CVA are not detected until the clinical symptoms are obvious in the middle and late stages, missing the optimal treatment time. FeNO combined with pulmonary function tests were used to predict the occurrence of small airway lesions, and precise medication was conducive to the rehabilitation of airway and pulmonary function and prognosis in children with CVA. In this study, we also found that the severity of CVA was positively correlated with FeNO and negatively correlated with small airway parameters, so the improvement rate of the 2 indicators can be used to determine the degree of recovery of CVA in clinical practice. It also can be used as an observation index for the effectiveness of drug treatment.

Our study has several limitations, which should be noted. First, the age range used in this study is small and the cases included are all Asian children, which may not be applicable to other regions. Second, our study did not include allergy parameters (e.g., IgE, eosinophil count), which may affect the generalizability of the results, and allergy testing should be performed in future studies including patients with CVA. In the future, multicenter and large-sample studies combining allergy parameters and dynamic monitoring are needed to optimize the diagnostic process.

## 5. Conclusion

In summary, FeNO and pulmonary function indexes (MMEF, MEF75, MEF50, MEF25) are closely related to the occurrence and development of CVA. Compared with FeNO, pulmonary function indexes had higher sensitivity in the diagnosis of CVA. FeNO combined with pulmonary function indexes detection is helpful to further improve the diagnostic efficacy of CVA and reduce missed diagnosis and misdiagnosis.

## Author contributions

**Investigation:** Yina Qiao.

**Supervision:** Xiaozheng Duan.

**Methodology:** Minghang Yang, Xiaofang Zhang.

**Writing – original draft:** Minghang Yang.

**Writing – review & editing:** Xiaochun Feng.

## References

[1] Tang W Zhou J Miao L Shi G. Clinical features in patients of cough variant asthma with normal and high level of exhaled fractional nitric oxide. Clin Respir J. 2018;12:595–600. 27731932 10.1111/crj.12568

[2] Uryasjev MO Ponomareva IV Bhar M Glotov SI. The cough variant asthma. Ter Arkh. 2020;92:98–101. 10.26442/00403660.2020.03.000404 32598800

[3] Liang HW Yi F Chen YH Lai KF Jiang M. Epidemiology of chronic cough in China: current status and future perspective. Zhonghua Jie He He Hu Xi Za Zhi. 2022;45:100–6. 35000314 10.3760/cma.j.cn112147-20211104-00773

[4] Gao J Wu F Wu S Yang X. Inflammatory subtypes in classic asthma and cough variant asthma. J Inflamm Res. 2020;13:1167–73. 33376381 10.2147/JIR.S269795 PMC7765682

[5] Zhou X Zhang Y Liu L Feng X Zhang H. Therapeutic effect of acupuncture combined montelukast sodium on cough variant asthma in children: a protocol for systematic review and meta-analysis. Medicine (Baltim). 2021;100:e28048. 10.1097/MD.0000000000028048 PMC8702241 34941045

[6] Zhu H Yu X Hao C. The diagnostic value of the fractional exhaled nitric oxide for cough variant asthma in children. Zhonghua Jie He He Hu Xi Za Zhi. 2015;38:352–5. 26463486

[7] Soto-Ramos M Castro-Rodríguez JA Hinojos-Gallardo LC Hernández-Saldaña R Cisneros-Castolo M Carrillo-Rodríguez V. Fractional exhaled nitric oxide has a good correlation with asthma control and lung function in Latino children with asthma. J Asthma. 2013;50:590–4. 23617392 10.3109/02770903.2013.792349

[8] Zhou Z Zhao D Zhang H. Understanding parental self-medication with antibiotics among parents of different nationalities: a cross-sectional study. Glob Health Res Policy. 2021;6:42. 34696814 10.1186/s41256-021-00226-y PMC8543833

[9] Feng-Jia C Xin-Yan H Geng-Peng L Yang-Li L Can-Mao X. Validity of fractional exhaled nitric oxide and small airway function indices in diagnosis of cough-variant asthma. J Asthma. 2018;55:750–5. 28846444 10.1080/02770903.2017.1366509

[10] Liu L Liu W Liu C. Study on small airway function in asthmatics with fractional exhaled nitric oxide and impulse oscillometry. Clin Respir J. 2018;12:483–90. 27606596 10.1111/crj.12548

[11] Chung KF. Increasing utility of FeNO as a biomarker of type-2 inflammation in severe asthma. Lancet Respir Med. 2021;9:1083–4. 34181878 10.1016/S2213-2600(21)00170-3

[12] Fujimura M Ogawa H Nishizawa Y Nishi K. Comparison of atopic cough with cough variant asthma: is atopic cough a precursor of asthma? Thorax. 2003;58:14–8. 12511712 10.1136/thorax.58.1.14 PMC1746464

[13] Kubo T Tobe K Okuyama K. Disease burden and quality of life of patients with chronic cough in Japan: a population-based cross-sectional survey. BMJ Open Respir Res. 2021;8:e000764. 10.1136/bmjresp-2020-000764 PMC8011713 33785505

[14] Chen SY Fang ZK Fang S. Comparison of functional parameters of small airways between patients with typical asthma and cough-variant asthma. Nan Fang Yi Ke Da Xue Xue Bao. 2017;37:330–6. 28377348 10.3969/j.issn.1673-4254.2017.03.09 PMC6780436

[15] Song WJ Kim HJ Shim JS. Diagnostic accuracy of fractional exhaled nitric oxide measurement in predicting cough-variant asthma and eosinophilic bronchitis in adults with chronic cough: a systematic review and meta-analysis. J Allergy Clin Immunol. 2017;140:701–9. 28088474 10.1016/j.jaci.2016.11.037

[16] Sato S Saito J Sato Y. Clinical usefulness of fractional exhaled nitric oxide for diagnosing prolonged cough. Respir Med. 2008;102:1452–9. 18614345 10.1016/j.rmed.2008.04.018

[17] Yi F Chen R Luo W. Validity of fractional exhaled nitric oxide in diagnosis of corticosteroid-responsive cough. Chest. 2016;149:1042–51. 26836931 10.1016/j.chest.2016.01.006

[18] Lane C Knight D Burgess S. Epithelial inducible nitric oxide synthase activity is the major determinant of nitric oxide concentration in exhaled breath. Thorax. 2004;59:757–60. 15333851 10.1136/thx.2003.014894 PMC1747143

[19] Bao W Zhang X Lv C. The value of fractional exhaled nitric oxide and forced mid-expiratory flow as predictive markers of bronchial hyperresponsiveness in adults with chronic cough. J Allergy Clin Immunol Pract. 2018;6:1313–20. 29128336 10.1016/j.jaip.2017.09.026

[20] Chen LC Zeng GS Wu LL. Diagnostic value of FeNO and MMEF for predicting cough variant asthma in chronic cough patients with or without allergic rhinitis. J Asthma. 2021;58:326–33. 31820665 10.1080/02770903.2019.1694035

[21] Tian C Xiong S Li S. Impulse oscillometry in the diagnosis of cough variant asthma in children. BMC Pediatr. 2024;24:296. 38702638 10.1186/s12887-024-04749-4 PMC11067131

[22] Wheeler DS Wong HR Zingarelli B. Pediatric sepsis – Part I: “Children are not small adults!”. Open Inflamm J. 2011;4:4–15. 23723956 10.2174/1875041901104010004 PMC3665507

[23] Wang Y Zhao L Chen F. Diagnostic value of fractional exhaled nitric oxide and small airway function in differentiating cough-variant asthma from typical asthma. Can Respir J. 2021;2021:9954411. 34457097 10.1155/2021/9954411 PMC8397554

